# Short Mood and Feelings Questionnaire for screening children and adolescents for plastic surgery: cross-cultural validation study

**DOI:** 10.1590/1516-3180.2017.0036030517

**Published:** 2017-11-06

**Authors:** Eduardo Sucupira, Miguel Sabino, Edson Luiz de Lima, Gal Moreira Dini, Maria José Azevedo de Brito, Lydia Masako Ferreira

**Affiliations:** I MD. Master’s Student, Postgraduate Program on Translational Surgery, Universidade Federal de São Paulo (UNIFESP), São Paulo (SP), Brazil.; II MD, PhD. Associate Professor, Division of Plastic Surgery, Department of Surgery, Universidade Federal de São Paulo (UNIFESP), São Paulo (SP), Brazil.; III MD, MSc. Physician, Instituto Federal de Educação, Ciência e Tecnologia do Sul de Minas (IFSuldeMinas), Pouso Alegre (MG), Brazil.; IV (in memoriam) PhD. Adjunct Professor, Postgraduate Program on Translational Surgery, Universidade Federal de São Paulo (UNIFESP), São Paulo (SP), Brazil.; V PhD. Affiliate Professor, College of Health Science, Universidade do Vale do Sapucaí (UNIVÁS), Minas Gerais; Division of Plastic Surgery, Department of Surgery, Universidade Federal de São Paulo (UNIFESP), São Paulo (SP), Brazil.; VI MD, PhD. Full Professor, Division of Plastic Surgery, Department of Surgery, Universidade Federal de São Paulo (UNIFESP), São Paulo (SP), Brazil.

**Keywords:** Adolescent, Self-image, Surgery, plastic, Depression, Triage

## Abstract

**CONTEXT AND OBJECTIVE:**

Patient-reported outcome measurements assessing the emotional state of children and adolescents who seek plastic surgery are important for determining whether the intervention is indicated or not. The aim of this study was to cross-culturally adapt and validate the Short Mood and Feelings Questionnaire (child/adolescent and parent versions) for Brazilian Portuguese, test its psychometric properties and assess the emotional state of children and adolescents who seek plastic surgery.

**DESIGN AND SETTING:**

Cross-cultural validation study conducted in a plastic surgery outpatient clinic at a public university hospital.

**METHODS:**

A total of 124 consecutive patients of both sexes were selected between September 2013 and February 2014. Forty-seven patients participated in the cultural adaptation of the questionnaire. The final version was tested for reliability on 20 patients. Construct validity was tested on 57 patients by correlating the Short Mood and Feelings Questionnaire (child/adolescent and parent versions) with the Strengths and Difficulties Questionnaire and the Rosenberg Self-Esteem scale.

**RESULTS:**

The child/adolescent and parent versions of the Short Mood and Feelings Questionnaire showed Cronbach’s alpha of 0.768 and 0.874, respectively, and had good inter-rater reliability (intraclass correlation coefficient, ICC = 0.757 and ICC = 0.853, respectively) and intra-rater reliability (ICC = 0.738 and ICC = 0.796, respectively).

**CONCLUSIONS:**

The Brazilian-Portuguese version of the Short Mood and Feelings Questionnaire is a reproducible instrument with face, content and construct validity. The mood state and feelings among children and adolescents seeking cosmetic surgery were healthy.

## INTRODUCTION

Childhood and adolescence are difficult times because of the enormous pressure imposed by society on people to conform to arbitrary standards of physical appearance.^1^^,^^2^ Standards of beauty help to shape thoughts, which may lead to discrepancy between what is conceived as ideal and the actual personal reality and also to higher demand for plastic surgery.^3^

Physical and emotional changes during adolescence may lead to dissatisfaction with physical appearance.^4^^,^^5^ At present, adolescents tend to seek esthetic and surgical procedures influenced by their peers or to improve interpersonal relationships and increase their feelings of inclusion in a social group.^4^

According to the American Society of Plastic Surgeons, about 283,000 cosmetic plastic surgeries were performed on adolescents aged between 13 and 19 in 2012.^6^ In Brazil, there was an increase of 141% in the number of plastic surgical procedures performed on adolescents between 14 and 18 years.^7^^,^^8^ The most common cosmetic procedures sought by girls, both in Brazil and worldwide, are liposuction and breast augmentation, and by boys are gynecomastia and otoplasty for correction of prominent ears.^4^^,^^6^ According to some authors, improvement in physical appearance is directly associated with increased self-esteem and self-confidence among adolescents.^1^^,^^9^

Thus, indications for plastic surgery may help some adolescents who feel different and uncomfortable in their own body to break out of social isolation.^4^^,^^9^ In fact, plastic surgery leads to psychological changes by modifying the physical appearance, and therefore is considered to be a psychological intervention.^10^^,^^11^^,^^12^ Thus, its impact is not only esthetic but also, especially, psychosocial. It is known that esthetics produces individual and social wellbeing.^9^^,^^13^

Body dysmorphic concerns may result in social anxieties and emotional conflicts among children and adolescents.^1^^,^^2^ Moreover, the presence of physical characteristics and appearance differing from the cultural standard of beauty may trigger bullying, which in turn causes psychological disorders among vulnerable individuals. Thus, the perception of a defect or flaw in physical appearance may contribute towards development of a mental disorder in individuals with neurobiological vulnerability and psychological fragility.^3^

Depression is the most common psychological disorder in contemporary society,^14^ with a prevalence of 2% among children and 4% to 8% among adolescents.^14^^,^^15^ The World Health Organization reported that depression is the most common disorder among children and adolescents between 10 and 19 years of age and is the predominant cause of disability in both genders. Suicide is one of the three leading causes of death in this age group.^16^ Mental health problems during childhood and adolescence are common and may be associated with various difficulties, including behavioral, emotional, social and academic functioning problems, thus affecting the development and use of potential resources.^17^

Excessive concern regarding appearance may conceal psychopathological states that are not always easily identified and may lead to iatrogenic and medico-legal problems if neglected.^18^ Thus, validation of patient-reported outcome measurements can help in rapidly screening and identifying depression among children and adolescents, since psychological disorders may not only affect their emotional, social and academic life,^19^ but also influence patient satisfaction with the results of surgery.^20^^,^^21^

## OBJECTIVE

To translate, culturally adapt and validate the Short Mood and Feelings Questionnaire (child/adolescent and parent versions)^22^ for Brazilian Portuguese; to test the psychometric properties, reproducibility and validity of the instrument; and to assess the emotional state of children and adolescents who seek plastic surgery.

## METHODS

This study was approved by the Institutional Research Ethics Committee of Universidade Federal de São Paulo (approval number 32664) and was conducted in accordance with the Brazilian Ethical Review System for research involving human beings. It also conformed to the 1964 Declaration of Helsinki and its subsequent amendments. Written informed consent was obtained from all patients and their parents or legal representatives after the procedures had been fully explained to them and prior to their inclusion in the study. Patient anonymity was assured. This study was conducted between September 2013 and February 2014.

Patients were consecutively recruited at the Plastic Surgery Outpatient Clinic of the “Jesus” Municipal Hospital and Barra Day Hospital in Rio de Janeiro (Brazil). The parents or legal representatives of the patients also participated in the study. The eligibility criteria were that patients of both sexes, aged between 8 and 17 years, showing preoccupation with physical appearance associated with subjective distress, and who were seeking plastic surgery, could be included. The exclusion criteria were lack of ability to understand the interview questions and presence of psychotic disorders.

A convenience sample (non-probability sample) of all consecutive patients who met the study criteria was selected to participate in the study. We assessed the highest possible number of eligible patients during the study period; none declined participation. Out of the 124 patients admitted, 47 participated in the cultural adaptation of the scale, 20 were included in the reliability analysis on the final version of the instrument and 57 participated in the construct validity assessment. The construct validity was assessed through correlating the Short Mood and Feelings Questionnaire, in its child/adolescent and parent versions, with the Strengths and Difficulties Questionnaire and the Rosenberg Self-Esteem scale. The participants in each phase were not included in the other phases of the study.

The number of patients participating in the cultural adaptation, reliability and validity phases was similar to that used in previous studies evaluating the psychometric properties of social construct measurements in plastic surgery populations,^23^^,^^24^^,^^25^^,^^26^^,^^27^^,^^28^^,^^29^ and was in accordance with the methodology internationally accepted and used for translation, cultural adaptation and validation of instruments.^30^^,^^31^^,^^32^^,^^33^^,^^34^ According to Sapnas and Zeller,^34^ the traditional protocol for determination of an adequate sample size based on power analysis is not appropriate for studies assessing the psychometric properties of social construct measurements; a total sample size of 50 subjects or more is adequate for representing the study population.^34^

### The instrument

The present study was conducted after Dr. Angold, the author of the original version of the Short Mood and Feelings Questionnaire, granted permission to translate, culturally adapt and validate the instrument for Brazilian Portuguese.

The Short Mood and Feelings Questionnaire^22^ is derived from the Mood and Feelings Questionnaire,^35^ which was developed to assess depressive signs and symptoms among children and adolescents between 8 and 17 years of age.^36^^,^^37^

The Short Mood and Feelings Questionnaire is a brief self-report instrument for screening for depressive symptoms and for assessing moods and feelings among children and adolescents; it is also available in a parent version. Thirteen items involving affective and cognitive components are rated on a scale from 0 to 2, where 0 indicates no symptom and 2 indicates depressive symptoms. The total score is calculated as the sum of ratings for the 13 items, with higher scores indicating mental health impairment of greater severity.

### Translation

The original version of the Short Mood and Feelings Questionnaire was translated from English into Brazilian Portuguese by two independent translators. Only one of the translators was informed about the objectives of the study, so as to obtain a conceptual rather literal translation of the scale.^30^ Both translations were evaluated by a multidisciplinary group composed of two plastic surgeons, a psychologist and an anesthesiologist. All items were checked for possible mistakes made during the translation and were evaluated for content validity. A Brazilian Portuguese consensus version of the questionnaire was then obtained by combining elements from both translations. The consensus version was adequately adapted for linguistic context and care was taken to preserve all essential characteristics of the original instrument. Idiomatic, semantic, conceptual and cultural equivalences were considered during the translation phase.

Next, the consensus version was back-translated into English by two independent translators who did not have any knowledge about the original questionnaire or purpose of the study. Both back-translated versions were evaluated and compared with the original questionnaire by the same multidisciplinary group, to check for possible errors made during back-translation. A consensus back-translated version was created and compared with the original English version, and minor differences were resolved by discussion. This analysis resulted in development of consensus version 1 of the Short Mood and Feelings Questionnaire in Brazilian Portuguese, which was appropriately adapted to the linguistic and cultural context of the target population, while maintaining all the essential characteristics of the original questionnaire in English.

### Cultural adaptation or pretesting

Version 1 of the questionnaire was administered to 20 patients and their respective parents, who were interviewed separately, to test for possible failures of the respondents to comprehend the items. After these patients had given informed consent for their participation, they were given the opportunity to express their comprehension of the questionnaire and suggest any changes they considered necessary. All patients and parents understood that the questionnaire items related to emotional state.

The interview data were collected and evaluated by the multidisciplinary team, and then version 2 of the scale was created, including adaptations that were necessary for patients and parents to properly understand all items. When patients failed to understand the meaning of an item, the question was reworded, while always maintaining the same semantic concept, so that the essential structure of the instrument was unchanged.

Version 2 of the scale was then administered to 27 different patients and their respective parents. The final version was obtained when patients, translators and healthcare professionals reached a consensus (Appendix 1).

### Psychometric evaluation

After translation and cultural adaptation, the final version was tested for internal reliability and for face, content and construct validity, on 20 and 57 target patients and parents, respectively.

### Reliability

Cronbach’s alpha (*α*) was used to evaluate the internal consistency and reliability of the instrument. It indicates the degree to which a set of items measures a single latent construct, thus determining the internal consistency or average correlation of items in a survey instrument and estimating its reliability.

Cronbach’s alpha ranges from 0 to 1. Alpha values greater than 0.7 indicate acceptable to high reliability.^38^^,^^39^ When the overall Cronbach’s alpha value is low (< 0.7), an item-by-item analysis should be carried out to evaluate whether an item should be excluded from the scale to increase the consistency of the instrument. The item-by-item analysis is performed by observing the correlation of each item with the other items of the instrument (item-total correlation) and by calculating “alpha if item deleted” for each item. If the item-total correlation is low and the alpha value if item deleted is higher than the overall alpha, it may be appropriate to remove this item from the scale.

Test-retest reliability (reproducibility) is the ability of an instrument to produce stable or similar results from repeated administration when no change in the patients’ characteristics has occurred.^33^ Studies have reported retesting as early as a few hours after baseline testing.^24^^,^^25^^,^^26^^,^^27^^,^^28^ The longer the time that elapses is, the lower the measured reliability will be, and the more likely it will be that knowledge or attitudes have in fact changed.^40^ The instrument was tested for test-retest reliability (reproducibility) in three interviews conducted by two independent interviewers. Twenty patients and parents were interviewed by investigator 1 and the interview was repeated by investigator 2 three hours later, on the same day. After two weeks, the instrument was administered to the same patients and parents by investigator 1 only. Inter and intra-rater reliability analyses were performed. This phase of testing is used to verify the precision of the instrument in measuring the properties for which it was designed.^31^^,^^32^

Statistical analysis on test-retest reliability was performed using the intraclass correlation coefficient (ICC) and Pearson’s correlation coefficient (r).

### Validity

In this study, face validity was determined through a consensus reached by the multidisciplinary group responsible for the Brazilian version of the questionnaire during its cultural adaptation.

Content validity is defined as the degree to which items are representative of the construct of interest. The content validity of the instrument was examined in each phase of the study by the multiprofessional group and determined through reaching a consensus.

Construct validity was tested on 57 patients and respective parents. This is the process in which the correlation of a measurement with other variables is tested for theoretical consistency. Construct validity was tested by comparing the Short Mood and Feelings Questionnaire with scales that are considered to be associated with mood and feelings, using convergent and divergent validity analyses.

Convergent validity refers to the degree to which two measurements of constructs that theoretically should be related are in fact related. Assessment of convergent validity does not require use of a gold standard. It was measured by studying the correlations between domains of the child/adolescent and parent versions of the Short Mood and Feelings Questionnaire and the child and parent versions of the Strengths and Difficulties Questionnaire.^17^^,^^41^ The Strengths and Difficulties Questionnaire has 25 items grouped into five subscales (emotional symptoms, conduct problems, hyperactivity-inattention, peer problems and prosocial behavior subscales) that assess positive and negative attributes of children and adolescents between 4 and 16 years of age. Higher scores on the prosocial behavior subscale reflect strengths, whereas higher scores on the other four subscales reflect difficulties. The instrument is available in three versions (child, parents and teachers).^17^ The correlation between the Short Mood and Feelings Questionnaire and the Strengths and Difficulties Questionnaire was tested using Pearson’s linear correlation.

Divergent validity demonstrates that the construct of interest (e.g. depression) is different from other constructs that might be present in the study (e.g. loss of self-esteem). Assessment of divergent validity does not require use of a gold standard. Divergent validity was determined by comparing scores on the Short Mood and Feelings Questionnaire, in its child/adolescent and parent versions, with scores on the Rosenberg Self-Esteem scale,^23^ using Pearson’s linear correlation. The Rosenberg Self-Esteem Scale is a 10-item measurement of self-esteem distributed over two domains: self-confidence and self-deprecation. The total score ranges from 0 to 30, where 0 indicates the highest level of self-esteem and 30 indicates the lowest level of self-esteem.

The Kolmogorov-Smirnov test was used to test the data for normal distribution. The Wilcoxon test was performed to evaluate differences in mean scores between the child/adolescent and parent versions of the Short Mood and Feelings Questionnaire, because the data were not distributed normally. Student’s t test for independent samples was used for comparisons of mean scores in the child/adolescent version of the Short Mood and Feelings Questionnaire, between age groups.

To evaluate the responsiveness of the Short Mood and Feelings Questionnaire, floor and ceiling effects were considered to be present if more than 10% of the respondents achieved the lowest or highest possible score, respectively.

The IBM Statistical Package for the Social Sciences, version 20.0 for Windows (IBM Corp., Armonk, NY, USA), and the Stata 12 software (Stata Corp, College Station, Texas, USA) were used for data analysis. All statistical tests were performed at a significance level of 5% (P < 0.05). Data were expressed as mean ± standard deviation (SD).

## RESULTS

**Figure 1.**
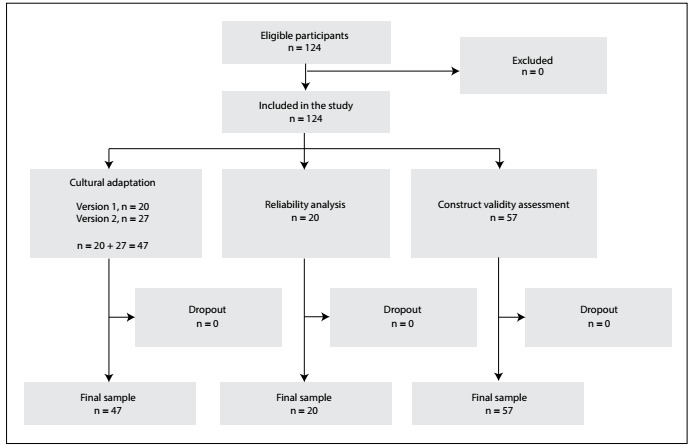
Flow diagram showing the initial recruitment and final sample of patients.

A convenience sample of 124 consecutive patients of both sexes was selected to participate in the study. No patient declined to participate. The flow diagram showing the initial recruitment and the final sample of patients is shown in Figure 1.

Overall, most patients 63.7% (n = 79) were boys; 48.4% (n = 60) were Caucasians; 86.3% (n = 107) had completed their primary education; the mean age was 12.1 ± 2.5 years; and 91.9% (n = 114) of the legal guardians who completed the parent version of the Short Mood and Feelings Questionnaire were the child’s natural parents (Table 1).

**Table 1.**
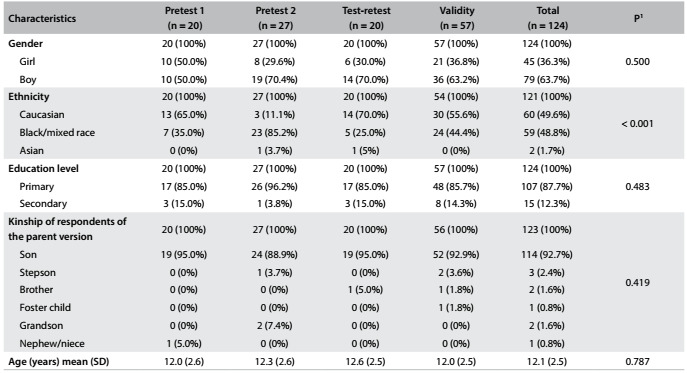
Characteristics of the children and adolescents in each phase of the study

^1^Fisher’s exact test or analysis of variance. SD = standard deviation.

### Cultural adaptation or pretesting

The purpose of the cultural adaptation or pretest was to evaluate whether the items of the translated instrument were clearly formulated. Thus, the 47 patients who participated in the pretest were not included in the statistical analysis.

Version 1 of the questionnaire was administered to 20 patients and respective parents. All the respondents understood that the items were about emotional states relating to mood and feelings. However, 20% (n = 4) of the children and adolescents and 15% (n = 3) of the parents did not understand the term “restless” (“inquieto” in Brazilian Portuguese) in item 4 (version 1), so the term was changed to “agitated” (“agitado” in Brazilian Portuguese) in both the child/adolescent and the parent version of the instrument.

The Short Mood and Feelings Questionnaire version 2 was then applied to another 27 children and adolescents and their parents, and the cross-cultural equivalence of the scale was retested. The patients and their parents had no doubts about the questionnaire items and found the instrument easy to understand. The mean time taken to answer the questionnaire was five minutes.

### Questionnaire scores

The mean scores on the Short Mood and Feelings Questionnaire, in the child/adolescent version (n = 77) and the parent version (n = 77) were 6.1 ± 4.4 and 6.9 ± 5.6, respectively. This showed that although the patients were dissatisfied with their physical appearance, they were mentally healthy. No significant difference was observed between scores from the child/adolescent and parent versions of the Short Mood and Feelings Questionnaire (P = 0.407; Wilcoxon test), and only a low correlation was found between the two versions of the instrument (r = 0.268; P = 0.019).

No significant age-related differences in scores from the child/adolescent version of the Short Mood and Feelings Questionnaire were found between children up to 11 years of age and those 12 years and older (P = 0.139; Student’s t test), thus showing that age had no impact on the degree of body dissatisfaction.

### Internal consistency

The child/adolescent version of the Short Mood and Feelings Questionnaire (n = 77) showed acceptable internal consistency (*α* = 0.768). All items contributed to the internal consistency of the scale, except for item 4, which showed an *α* of -0.086, thus indicating almost complete absence of correlation of this item with the others. Deletion of item 4 (I felt very agitated) increased the internal consistency (*α* = 0.808), as shown in Table 2. The parent version of the Short Mood and Feelings Questionnaire (n = 77) showed good internal consistency (*α* = 0.874), with all items contributing favorably towards the internal consistency of the scale (Table 2).

**Table 2.**
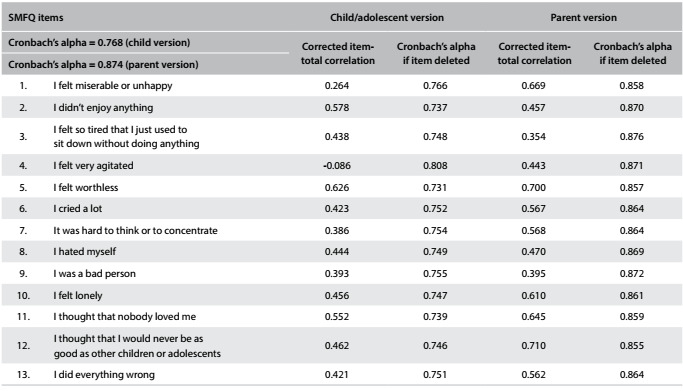
Internal consistency analysis. Statistical summary of scores from the child/adolescent and parent versions of the Short Mood and Feelings Questionnaire (SMFQ) (n = 77)

No floor or ceiling effect was present for the child/adolescent and parent versions of the Short Mood and Feelings Questionnaire, thus showing that both versions had good responsiveness.

### Test-retest reliability

Inter-rater and intra-rater reliability were investigated in a sample of 20 patients and respective parents. The child/adolescent version of the Short Mood and Feelings Questionnaire demonstrated good inter-rater reliability (r = 0.808; ICC = 0.757; P < 0.001) and intra-rater reliability (r = 0.801; ICC = 0.738; P < 0.001), as seen in Table 3. The parent version of the Short Mood and Feelings Questionnaire also had good inter-rater reliability (r = 0.894; ICC = 0.853; P < 0.001) and intra-rater reliability (r = 0.816; ICC = 0.796; P < 0.001), as listed in Table 4.

**Table 3.**
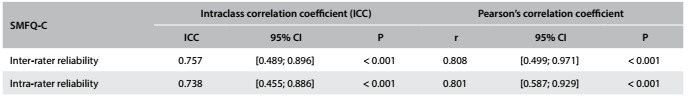
Inter and intra-rater reliability of the child/adolescent version of the Short Mood and Feelings Questionnaire (SMFQ-C) (n = 20)

CI = confidence interval; ICC = intraclass correlation coefficient; r = Pearson’s correlation coefficient.

**Table 4.**
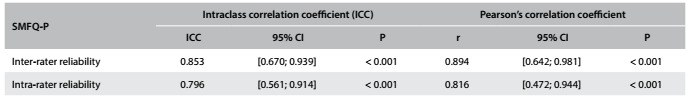
Inter and intra-rater reliability of the parent version of the Short Mood and Feelings Questionnaire (SMFQ-P) (n = 20)

CI = confidence interval; ICC = intraclass correlation coefficient; r = Pearson’s correlation coefficient.

### Construct validity

Construct validity was evaluated in a sample of 57 adolescent patients and their parents. There was a low correlation between the child/adolescent version of the Short Mood and Feelings Questionnaire and the child version of the Strengths and Difficulties Questionnaire (r = 0.295; P = 0.044), and a moderate correlation between the child/adolescent version of the Short Mood and Feelings Questionnaire and the Rosenberg Self-Esteem scale (r = 0.495; P < 0.001).

A moderate correlation was found between the parent version of the Short Mood and Feelings Questionnaire and the parent version of the Strengths and Difficulties Questionnaire (r = 0.581; P < 0.001).

## DISCUSSION

The Short Mood and Feelings Questionnaire^22^ is a brief self-report questionnaire that captures specific information about depressive symptoms and can serve as a decision-support system for selecting children and adolescents as candidates for plastic surgery. In contrast to the Diagnostic and Statistical Manual of Mental Disorders, fifth edition (DSM-5), which is intended for use by psychiatrists in making diagnoses of mental disorders such as depression, the Short Mood and Feelings Questionnaire is a brief, easy-to-use, objective screening tool that can be administered by healthcare professionals in general, thus allowing symptom tracking.

General guidelines for cross-cultural adaptation of quality-of-life instruments were followed to ensure quality in the cross-culturally adapted Brazilian version of the Short Mood and Feelings Questionnaire (Appendix 1). Patients and healthcare professionals who were experienced in management of plastic surgery patients participated in the evaluation of this instrument.^30^

The Brazilian Portuguese version of the Short Mood and Feelings Questionnaire was validated in a population sample of esthetic surgery patients (n = 77). The most common complaint among these children and adolescents was prominent ears, as reported by other researchers,^4^^,^^6^^,^^42^^,^^43^^,^^44^ and the main motivation for seeking otoplasty was marked psychological and social distress, a finding consistent with previous studies.^42^^,^^44^ The mean age of 12 years was similar to what was found by Rhew et al.^44^ in a validation study on the Short Mood and Feelings Questionnaire.

The 47 patients who were interviewed to assess the cross-cultural equivalence of the translated Short Mood and Feelings Questionnaire^32^ found that the instrument was easy to understand. The mean time taken to answer to the questionnaire was five minutes.

The instrument showed good internal consistency (child/adolescent version, *α* = 0.76; parent version, *α* = 0.87), compared with the original instrument (child/adolescent version, *α* = 0.85; parent version, *α* = 0.87),^22^ as well as good inter-rater reliability (child/adolescent version, ICC = 0.76; parent version, ICC = 0.85), compared with the original scale (child/adolescent version, ICC = 0.73; parent version, ICC = 0.75),^35^ and intra-rater reliability (child/adolescent version, ICC = 0.73; parent version, ICC = 0.79).

Item 4 of the Short Mood and Feelings Questionnaire had to be changed in both the child/adolescent and parent versions, and negatively affected the internal consistency of the scale. Similarly, Lundervold et al.^45^ found excellent internal consistency for all items of the Short Mood and Feelings Questionnaire, except for item 4. Sharp et al.^46^ reported that items 3, 4 and 7 had no discriminatory power, especially for high scores, but contributed towards screening for patients reporting low scores from the Short Mood and Feelings Questionnaire. The variables of restlessness and tiredness, which are assessed in these items, may be related to changes to sleep-wake pattern during adolescence, resulting from physiological and psychological factors.^47^ In this study, the lowest scores reported were for items 3, 4 and 7, which assessed restlessness, tiredness and concentration problems, respectively, which are symptoms of depression.^45^ The low scores indicated that despite the distress with their physical appearance, the patients were mentally healthy.

The validity of the instrument was tested by comparing the Short Mood and Feelings Questionnaire with similar tools.^33^ The Brazilian versions of the Strengths and Difficulties Questionnaire and the Rosenberg Self-Esteem scale are cross-culturally adapted and validated instruments that measure aspects of mental health. The moderate and low correlations of the Short Mood and Feelings Questionnaire with the Rosenberg Self-Esteem scale and the Strengths and Difficulties Questionnaire, respectively, indicated that the study participants were mentally healthy. The children and adolescents reported a mean score of 9.9 ± 3.9 on the Rosenberg Self-Esteem scale, thus indicating good self-esteem, which is a mental health indicator. Individuals with good self-esteem are less likely to have depression.^12^

The fact that the Strengths and Difficulties Questionnaire assesses various emotional problems and is not specific to depressive symptoms may explain the low correlation between the two questionnaires. The moderate correlation between the parent version of the Short Mood and Feelings Questionnaire and the parent version of the Strengths and Difficulties Questionnaire suggested that parents provided a more rigorous evaluation of both the mental condition of their children^47^ and their own subjectivity. However, the correlation between parent and child perception showed that although there was an affective bond between them, there was also independence of affections and presence of individuality. Parents can be a relevant source of information.^9^ Children often cannot adequately express their feelings about physical issues that may be affecting them emotionally. This highlights the importance of validating the child/adolescent and parent versions of the Short Mood and Feelings Questionnaire.

Although the Strengths and Difficulties Questionnaire assesses emotional problems and the Rosenberg Self-Esteem Scale measures self-deprecation, these instruments were not designed to specifically measure depressive signs and symptoms among children and adolescents. Thus, the Short Mood and Feelings Questionnaire is a valuable screening tool for rapid and simple detection of mental health impairment among children and adolescents, and may provide support for selecting patients for plastic surgery procedures.

This study was conducted mostly on boys and the main motivation for seeking plastic surgery was prominent ears. This is a limitation on the generalization of the results. Further studies are necessary to test the performance of the Short Mood and Feelings Questionnaire in different populations of children and adolescents.

## CONCLUSIONS

The Short Mood and Feelings Questionnaire was translated, culturally adapted and validated for Brazilian Portuguese and was named the “Short Mood and Feelings Questionnaire-Escola Paulista de Medicina/UNIFESP” or SMFQ-EPM/UNIFESP. It is a reliable instrument, showing face, content and construct validity. The Short Mood and Feelings Questionnaire indicated that the mood state and feelings of children and adolescents seeking cosmetic surgery were healthy.
